# Clarithromycin as an immunomodulator in sepsis: still a (IN)CLASS act

**DOI:** 10.1186/s13054-022-04104-y

**Published:** 2022-08-05

**Authors:** Timothy Arthur Chandos Snow, Antonio Cesar, Mervyn Singer, Nishkantha Arulkumaran

**Affiliations:** Bloomsbury Institute of Intensive Care Medicine, University College London, Gower Street, London, WC1E 6DH UK

Dear Editor,

We read with great interest the INCLASS study by Karakike et al [1]. We commend the authors on conducting a clinical trial providing mechanistic insights, underpinned by a sound scientific rationale. While the primary objective, 28-day mortality, was unaffected by addition of clarithromycin to a beta-lactam in the management of community-acquired pneumonia, the authors report a significant reduction in sepsis recurrence associated with increased monocyte HLA-DR. The lack of mortality benefit associated with clarithromycin use is consistent with a previous study from the authors in patients with ventilator-associated pneumonia [2].

Persistent downregulation of monocyte HLA-DR expression, and lymphopenia are characteristic of sepsis-induced immunosuppression. The authors suggest a four-day course of clarithromycin may expedite recovery of monocyte HLA-DR expression by day- 10 which may, in turn, be responsible for reduced recurrence of sepsis. It would be intriguing to know if monocyte co-stimulatory molecule CD86 increased in tandem, as reported by the authors in a previous study [3]. Assessment of effector cell receptor expression (e.g., T-lymphocyte CD28 and CTLA4) would provide greater insight.

Transcriptomics were performed from total RNA isolated from whole blood, in which monocytes constitute a small proportion of the leukocyte population. Whether whole blood transcriptomic data reflect changes specifically in monocytes is questionable. This may explain why RNA sequencing data did not reveal differences in pathways regulating monocyte HLA-DR, even though HLA-DR is typically regulated at the transcriptional level via Class II transactivator (CIITA) [4]. Intuitively, a reduction in HLA-DR expression would be expected as clarithromycin inhibits protein translation via bacterial ribosomal inhibition, with human ribosomes affected at higher concentrations [5].

Although clarithromycin use was associated with a significantly lower day- 28 sepsis recurrence (67.9% vs. 30.4%), acute kidney injury was in fact, more common in patients receiving clarithromycin and mortality rates (at both 28 and 90 days) were similar between groups.

Finally, it is worth commenting on antimicrobial resistance issues. Antimicrobial resistance is now endemic in many parts of the world. The SENTRY Antimicrobial Surveillance Program, collected between 2015 and 2017, reported that 32.4% of *S. pneumoniae* isolates were resistant to azithromycin. The authors report that the pathogens identified in their patient population were already highly resistant (multi-drug resistant 24.4%; extremely drug-resistant 47.4%; pan-drug resistant 7.7%) on study enrollment, but do not provide data on macrolide resistance. Additionally, the emergence of macrolide-resistant organisms following clarithromycin treatment is worthy of reporting.

Whilst this trial may not have achieved its primary clinical outcome, it adds to our understanding of macrolide-induced immunomodulation. Identification of the optimal dose, time, duration and patient cohort to benefit from macrolide-induced immune modulation are yet to be realised.

## Data Availability

Not applicable.
